# Perceived fatalism regarding possible COVID-19 infection and its association with self-reported comorbidities in 12 Latin American countries

**DOI:** 10.1186/s12879-026-14042-7

**Published:** 2026-07-24

**Authors:** Christian R. Mejia, Camilo Vega-Useche, Víctor Serna-Alarcón, Jamil Cedillo-Balcázar, Scherlli E. Chamorro-Espinoza, Jaime A. Yáñez, Neal M. Davies, Dalia Useche-Villamizar

**Affiliations:** 1 Universidad de Huánuco, Huánuco, Peru; 2 Fundación Santa Fe de Bogotá, Bogotá, Colombia; 3 Universidad Pedagógica y Tecnológica de Colombia, Tunja, Colombia; 4 Universidad Privada Antenor Orrego, Piura, Peru; 5 Hospital de la Amistad Perú Corea Santa Rosa II-2, Piura, Peru; 6 Dirección Provincial de Salud de Loja, Ministerio de Salud Pública, Loja, Ecuador; 7 Asociación Médica de Investigación y Servicios en Salud, Lima, Peru; 8 Escuela de Posgrado, Universidad Internacional Iberoamericana, Campeche, Mexico; 9 University of Alberta, Edmonton, Canada

**Keywords:** COVID-19, Fatalism, Comorbidity, Mental health, Latin America, Cross-sectional study

## Abstract

**Introduction:**

The COVID-19 pandemic has had substantial mental health consequences worldwide. However, evidence on perceived fatalism regarding possible COVID-19 infection, particularly in relation to self-reported comorbidities, remains limited in Latin America.

**Objective:**

To assess perceived fatalism regarding possible COVID-19 infection and its association with self-reported comorbidities among participants recruited in 12 Latin American countries.

**Methods:**

This multicenter analytical cross-sectional study used a non-probability online snowball sampling strategy from June to August 2020. The study included 6,530 participants from Peru, Chile, Paraguay, Mexico, Colombia, Bolivia, Panama, Ecuador, Costa Rica, El Salvador, Honduras, and Guatemala. Perceived fatalism was assessed using five items from a previously developed COVID-19 fatalism scale. The five-item overall score was the primary measure, whereas domain-specific analyses were considered exploratory because of their lower reliability. Self-reported respiratory disease, cardiac disease, diabetes mellitus, obesity, and other illnesses were analyzed as exposure variables. Adjusted prevalence ratios (aPRs) and 95% confidence intervals (CIs) were estimated using generalized linear models from the Poisson family with log link and robust variance.

**Results:**

The median age was 22 years (IQR: 19–28), 58.8% of participants were female, 25.3% reported at least one comorbidity, and 55.0% of the sample was recruited in Peru. The five-item fatalism scale showed acceptable internal consistency (Cronbach’s alpha = 0.707; McDonald’s omega/Raykov’s rho = 0.721). In adjusted models, high overall fatalism was more prevalent among participants reporting respiratory disease (aPR 1.17, 95% CI 1.05–1.30), cardiac disease (aPR 1.25, 95% CI 1.08–1.44), diabetes mellitus (aPR 1.32, 95% CI 1.09–1.61), obesity (aPR 1.23, 95% CI 1.10–1.37), and other illnesses (aPR 1.26, 95% CI 1.15–1.39). Men had a lower prevalence of high overall fatalism (aPR 0.88, 95% CI 0.83–0.94). Sensitivity analyses yielded materially similar estimates.

**Conclusions:**

In this non-probabilistic sample from 12 Latin American countries, self-reported comorbidities were associated with a modestly higher prevalence of perceived fatalism regarding possible COVID-19 infection. These findings should be interpreted as exploratory and hypothesis-generating, given the cross-sectional design, self-reported exposures, potential residual confounding, and the young, Peru-predominant, non-probability sample. They should not be interpreted as nationally representative estimates or as evidence of causal relationships.

**Supplementary Information:**

The online version contains supplementary material available at https://doi.org/10.1186/s12879-026-14042-7.

## Introduction

The COVID-19 pandemic has had a diverse range of consequences worldwide. Beyond the highly virulent acute respiratory disease and the associated mortality, there is evidence of increased psychological disorders and adverse mental health effects [1–3]. This may be partly due to the initial uncertainty surrounding the pandemic’s impact. Although previous viral outbreaks, such as severe acute respiratory syndrome (SARS-CoV) in 2003 and Middle East respiratory syndrome (MERS) in 2012, affected multiple regions [4], none had the scale of impact observed with COVID-19. Furthermore, the pandemic’s effects on the global economy and the social restriction measures implemented to reduce viral transmission have significantly influenced population mental health [5].

These circumstances contributed to the emergence of psychiatric symptoms during the pandemic. Reported conditions include post-traumatic stress disorder, anxiety, depression, and feeding disorders [6, 7], highlighting the critical importance of mental health globally. While it is necessary to evaluate these symptoms individually, it is also essential to consider their potential consequences, as they may lead individuals to make fatal decisions, including suicide [8].

Perceived fatalism, which is defined as the belief that events are predestined and beyond one’s control, is positively associated with depression and maladaptive coping strategies [9]. For example, studies reported that in Peru, individuals experienced depression, feared death, or believed the pandemic could signify the end of the world, with some reporting potential for fatal actions [10, 11].

Assessing perceived fatalism regarding the possibility of COVID-19 infection is therefore crucial, particularly in Latin America, where the disease disproportionately affects patients with prevalent comorbidities such as diabetes, hypertension, and obesity [12, 13]. Yet, many countries in the region still lack such an evaluation. Given the substantial burden of COVID-19 in Latin America and the increased clinical vulnerability of individuals with chronic conditions, this study aimed to assess perceived fatalism regarding possible COVID-19 infection and its association with self-reported comorbidities among participants recruited in 12 Latin American countries.

## Methods

We conducted a multicenter, analytical, cross-sectional study from June to August 2020 in 12 Latin American countries: Peru, Chile, Paraguay, Mexico, Colombia, Bolivia, Panama, Ecuador, Costa Rica, El Salvador, Honduras, and Guatemala. These countries were selected to represent some of the most affected regions in Latin America during the COVID-19 pandemic [14].

A non-probability snowball sampling strategy was used. The survey was administered online, and each local research team distributed the questionnaire through social networks, email, text messages, and direct invitations. Participants were also encouraged to share the survey with their contacts. A total of 6,530 participants were included in the analytical sample. Because recruitment was conducted online and through snowball sampling, the sample should not be interpreted as nationally representative of the participating countries. The resulting sample was predominantly young and likely overrepresented more digitally connected individuals. Country-specific sample sizes were markedly unequal, with Peru contributing more than half of the analytical sample.

Inclusion criteria were being aged 18 years or older, residing in one of the participating countries, providing electronic informed consent, and completing the online survey. Surveys with incomplete or inconsistent responses were excluded during data cleaning. Self-reported comorbidities were not used as an eligibility criterion; instead, they were analyzed as exposure variables. These included respiratory disease, cardiac disease, diabetes mellitus, obesity, and other illnesses. A composite variable indicating the presence of at least one self-reported comorbidity was also created for sensitivity analyses. Because previous COVID-19 diagnosis was self-reported and subject to potential misclassification, we conducted a sensitivity analysis restricted to participants reporting no previous COVID-19 diagnosis.

Perceived fatalism was assessed using five items from a previously developed COVID-19 fatalism scale [15]. The present analysis was restricted to the five items available in the multicountry survey dataset, which captured key perceived consequences of possible infection: infecting relatives or friends, hospitalization, depression, death, and the perception that the pandemic reflected the “end of the world”. Because the complete seven-item instrument was not administered, the present study did not attempt to reproduce or claim full validation of the original scale. The five-item score was therefore treated as a shortened study-specific measure derived from the previously developed instrument. Responses ranged from “completely disagree” to “completely agree”.

In the present sample, the shortened five-item measure showed acceptable internal consistency (Cronbach’s alpha = 0.707; McDonald’s omega/Raykov’s rho = 0.721). The five-item overall score was treated as the primary fatalism outcome because it showed the highest internal consistency and provided the broadest representation of the construct available in the dataset. The items were also grouped conceptually into two secondary domains: infection/hospitalization and consequences of contagion. The infection/hospitalization domain showed lower reliability using Cronbach’s alpha (α = 0.545), and omega could not be estimated because it contained only two items. The consequences domain showed Cronbach’s alpha = 0.642 and McDonald’s omega/Raykov’s rho = 0.662. Given these lower reliability coefficients, particularly for the two-item domain, domain-specific analyses were considered secondary and exploratory. Their estimates were interpreted with reduced confidence because measurement error may have attenuated or destabilized the observed associations.

The five fatalism items were summed to generate an overall score ranging from 5 to 25. High overall fatalism was defined as a score of 16–25, corresponding to an average response above the neutral category. This overall measure constituted the primary outcome. The infection/hospitalization domain included two items and was dichotomized as 8–10 versus 2–7, whereas the consequences domain included three items and was dichotomized as 9–15 versus 3–8. Analyses of these two domains were secondary and exploratory because their reliability was lower than that of the overall score.

Internal consistency of the shortened fatalism scale was assessed using Cronbach’s alpha and McDonald’s omega/Raykov’s rho. Crude and adjusted prevalence ratios (PRs and aPRs), 95% confidence intervals (CIs), and p-values were estimated using generalized linear models from the Poisson family with log link and robust variance. The main multivariable models were adjusted for sex, age, country of residence, and self-reported comorbidities, including respiratory disease, cardiac disease, diabetes mellitus, obesity, and other illnesses. Age was modeled as a continuous variable. Country of residence was included as a categorical variable, with Peru as the reference category because it contributed the largest number of participants.

Sensitivity analyses were conducted by additionally adjusting for educational attainment, replacing the individual comorbidity indicators with a composite variable indicating at least one self-reported comorbidity, excluding countries with fewer than 100 participants, and restricting the sample to participants reporting no previous COVID-19 diagnosis. Complete estimates from these analyses are presented in Supplementary Tables S1–S4. Statistical significance was defined at *p* < 0.05. All analyses were performed using Stata.

### Ethical considerations

This study was approved in 2020 by the Research Bioethics Committee of the Universidad Privada Antenor Orrego (Resolution Number 0237-2020-UPAO). Prior to completing the virtual questionnaire, participants provided informed consent electronically. All data were collected anonymously, in accordance with the ethical principles outlined in the Declaration of Helsinki.

## Results

Among the 6,530 participants included in the analytical sample, 3,840 (58.8%) were female and 2,690 (41.2%) were male. The median age was 22 years (interquartile range: 19–28 years). Comorbidities were reported as follows: respiratory diseases in 8.4% (*n* = 549), cardiac diseases in 3.7% (*n* = 239), diabetes mellitus in 1.6% (*n* = 103), obesity in 6.5% (*n* = 424), and other diseases in 9.2% (*n* = 600). Overall, 1,650 participants (25.3%) reported at least one of the assessed comorbidities, whereas 4,880 (74.7%) reported none. Baseline characteristics are presented in Table 1.

**Table 1:** Baseline characteristics of participants

Multicenter online survey in 12 Latin American countries, *n* = 6,530	
	*n* (%) or median (IQR)
**Overall**	
Total participants	6,530
**Sociodemographic characteristics**	
Age, median (IQR), years	22 (19–28)
Female	3,840 (58.8%)
Male	2,690 (41.2%)
**Self-reported comorbidities**	
Respiratory disease	549 (8.4%)
Cardiac disease	239 (3.7%)
Diabetes mellitus	103 (1.6%)
Obesity	424 (6.5%)
Other illness	600 (9.2%)
At least one self-reported comorbidity	1,650 (25.3%)
No assessed comorbidity reported	4,880 (74.7%)
**Country of residence**	
Peru	3,589 (55.0%)
Chile	748 (11.5%)
Paraguay	555 (8.5%)
Mexico	344 (5.3%)
Colombia	73 (1.1%)
Bolivia	344 (5.3%)
Panama	144 (2.2%)
Ecuador	272 (4.2%)
Costa Rica	99 (1.5%)
El Salvador	223 (3.4%)
Honduras	95 (1.5%)
Guatemala	44 (0.7%)

Values are presented as n (%) unless otherwise indicated. Percentages were calculated using the total analytical sample as the denominator. IQR: interquartile range. Comorbidities were self-reported and analyzed as exposure variables.

Table 2 presents the percentage of participants who selected “agree” or “completely agree” for each item of the shortened fatalism scale regarding possible COVID-19 infection. The most frequently endorsed item was the perception of being able to infect relatives or friends (69.7%), followed by the perception of possible hospitalization due to complications (43.2%) and depression if infected (39.1%). Lower proportions of participants agreed or completely agreed that they could die from infection (25.4%) or that the pandemic reflected how close the “end of the world” was (12.2%).

**Table 2:** Distribution of responses to the shortened fatalism scale. Participants selecting “agree” or “completely agree”, n = 6,530

**Item**	**Agree or completely agree**,** n (%)**
I think that, if I get the disease, I will infect my relatives/friends	4,553 (69.7%)
I think that I will be hospitalized due to a complication	2,818 (43.2%)
I think that, by getting this virus, I will be depressed	2,556 (39.1%)
I think that, by getting this virus, I could die	1,656 (25.4%)
I think that this evidences how close the “end of the world” is	794 (12.2%)

Values correspond to participants who selected “agree” or “completely agree” for each item. Percentages were calculated using the total analytical sample as the denominator.

The shortened five-item fatalism measure showed acceptable internal consistency (Cronbach’s alpha = 0.707; McDonald’s omega/Raykov’s rho = 0.721). The infection/hospitalization domain showed lower internal consistency (Cronbach’s alpha = 0.545), and omega could not be estimated because the domain included only two items. The consequences domain showed Cronbach’s alpha = 0.642 and McDonald’s omega/Raykov’s rho = 0.662. Accordingly, domain-specific analyses were considered exploratory, and their estimates should be interpreted with less confidence than the findings for the overall five-item score.

**Fig. 1:**
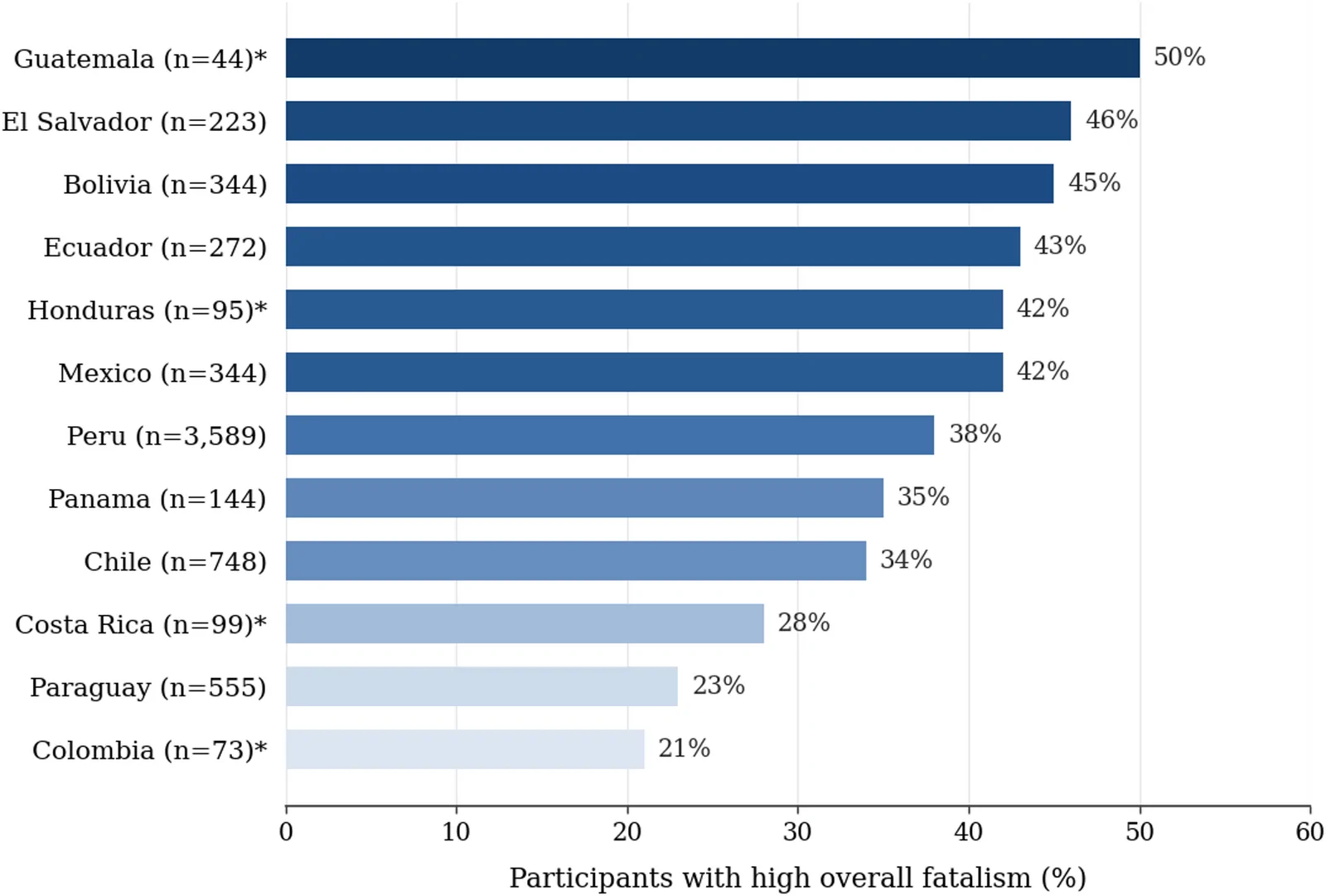
Percentage of participants with high overall fatalism regarding possible COVID-19 infection in the study sample from 12 Latin American countries. High overall fatalism was defined as a total score of 16–25 on the shortened five-item fatalism scale. Countries with fewer than 100 participants are marked with an asterisk. Percentages are descriptive estimates for the recruited sample and should not be interpreted as nationally representative prevalence estimates or as valid population-level comparisons between countries. Interpretation is limited by the non-probability online sampling strategy, the predominance of participants from Peru, and the markedly unequal country-specific sample sizes

As shown in Fig. 1, the proportion of participants with high overall fatalism varied descriptively among the country-specific subsamples. The observed proportions ranged from 21% in Colombia and 23% in Paraguay to 46% in El Salvador and 50% in Guatemala. These percentages are presented for descriptive purposes only and should not be interpreted as national prevalence estimates or as evidence of true differences between national populations. Recruitment was non-probabilistic, Peru contributed 55.0% of the total sample, and several countries had small and markedly unequal sample sizes.

As shown in Table 3, all self-reported comorbidities were associated with a higher prevalence of high overall fatalism. The strongest associations were observed for diabetes mellitus (aPR 1.32, 95% CI 1.09–1.61), other illnesses (aPR 1.26, 95% CI 1.15–1.39), cardiac disease (aPR 1.25, 95% CI 1.08–1.44), obesity (aPR 1.23, 95% CI 1.10–1.37), and respiratory disease (aPR 1.17, 95% CI 1.05–1.30). Male sex was associated with a lower prevalence of high overall fatalism (aPR 0.88, 95% CI 0.83–0.94), while age was not clearly associated with this outcome. In exploratory adjusted comparisons using Peru as the reference category, high overall fatalism was more prevalent in the recruited subsamples from Bolivia and El Salvador and less prevalent in those from Chile, Paraguay, Colombia, and Costa Rica. These country coefficients are sample-specific and should not be interpreted as national population contrasts because recruitment was non-probabilistic and country-specific sample sizes were markedly unequal.

**Table 3:** Factors associated with high overall fatalism regarding possible COVID-19 infection

Generalized linear model with Poisson family, log link, and robust variance			
	aPR	95% CI	*p*-value
**Sociodemographic characteristics**			
Male sex	0.88	0.83–0.94	< 0.001
Age, per year	1.00	1.00–1.00	0.190
**Self-reported comorbidities**			
Respiratory disease	1.17	1.05–1.30	0.003
Cardiac disease	1.25	1.08–1.44	0.002
Diabetes mellitus	1.32	1.09–1.61	0.004
Obesity	1.23	1.10–1.37	< 0.001
Other illness	1.26	1.15–1.39	< 0.001
**Country of residence**			
Peru	Reference	—	—
Chile	0.84	0.75–0.93	0.001
Paraguay	0.57	0.48–0.67	< 0.001
Mexico	1.09	0.95–1.24	0.204
Colombia	0.54	0.34–0.85	0.008
Bolivia	1.16	1.03–1.32	0.018
Panama	0.93	0.74–1.16	0.505
Ecuador	1.13	0.98–1.31	0.092
Costa Rica	0.71	0.52–0.97	0.031
El Salvador	1.23	1.06–1.42	0.006
Honduras	1.10	0.87–1.39	0.430
Guatemala	1.27	0.92–1.73	0.140

aPR: adjusted prevalence ratio; CI: confidence interval. The model was adjusted for sex, age, self-reported respiratory disease, cardiac disease, diabetes mellitus, obesity, other illness, and country of residence

High overall fatalism was defined as a total score of 16–25 on the shortened five-item fatalism scale

As shown in Table 4, respiratory disease (aPR 1.16, 95% CI 1.06–1.28), diabetes mellitus (aPR 1.22, 95% CI 1.03–1.46), obesity (aPR 1.15, 95% CI 1.03–1.28), and other illnesses (aPR 1.18, 95% CI 1.08–1.30) were associated with a higher prevalence of high concern about infection or hospitalization. Cardiac disease was not clearly associated with this outcome after adjustment (aPR 1.11, 95% CI 0.97–1.28). Age showed a small positive association, while male sex was not clearly associated with this domain. In exploratory adjusted comparisons using Peru as the reference category, high concern about infection or hospitalization was more prevalent in the recruited subsamples from Mexico, El Salvador, Honduras, and Guatemala and less prevalent in those from Chile, Paraguay, Colombia, and Panama. These country-specific coefficients should be interpreted cautiously because they reflect non-probability subsamples with markedly unequal sizes.

**Table 4:** Factors associated with high concern about infection or hospitalization

Generalized linear model with Poisson family, log link, and robust variance			
	aPR	95% CI	*p*-value
**Sociodemographic characteristics**			
Male sex	0.97	0.91–1.03	0.283
Age, per year	1.01	1.00–1.01	< 0.001
**Self-reported comorbidities**			
Respiratory disease	1.16	1.06–1.28	0.002
Cardiac disease	1.11	0.97–1.28	0.122
Diabetes mellitus	1.22	1.03–1.46	0.025
Obesity	1.15	1.03–1.28	0.013
Other illness	1.18	1.08–1.30	< 0.001
**Country of residence**			
Peru	Reference	—	—
Chile	0.69	0.62–0.78	< 0.001
Paraguay	0.62	0.53–0.72	< 0.001
Mexico	1.13	1.00–1.27	0.046
Colombia	0.61	0.42–0.91	0.014
Bolivia	1.02	0.90–1.16	0.715
Panama	0.63	0.48–0.83	0.001
Ecuador	1.10	0.96–1.25	0.193
Costa Rica	0.89	0.70–1.14	0.358
El Salvador	1.29	1.13–1.47	< 0.001
Honduras	1.25	1.03–1.52	0.023
Guatemala	1.46	1.15–1.85	0.002

aPR: adjusted prevalence ratio; CI: confidence interval. The model was adjusted for sex, age, self-reported respiratory disease, cardiac disease, diabetes mellitus, obesity, other illness, and country of residence.

High concern about infection or hospitalization was defined as a score of 8–10 on the two-item infection/hospitalization domain.

As shown in Table 5, all self-reported comorbidities were associated with a higher prevalence of high perceived consequences of contagion. The largest associations were observed for diabetes mellitus (aPR 1.51, 95% CI 1.25–1.84) and cardiac disease (aPR 1.35, 95% CI 1.17–1.56), followed by other illnesses (aPR 1.27, 95% CI 1.15–1.40), obesity (aPR 1.23, 95% CI 1.09–1.38), and respiratory disease (aPR 1.17, 95% CI 1.05–1.30). Male sex was associated with a lower prevalence of high perceived consequences of contagion (aPR 0.90, 95% CI 0.84–0.96), while age was not clearly associated with this outcome. In exploratory adjusted comparisons using Peru as the reference category, high perceived consequences of contagion were more prevalent in the recruited subsample from Bolivia and less prevalent in those from Chile, Paraguay, and Costa Rica. These coefficients should not be interpreted as nationally representative differences.

**Table 5:** Factors associated with high perceived consequences of contagion

Generalized linear model with Poisson family, log link, and robust variance			
	aPR	95% CI	*p*-value
**Sociodemographic characteristics**			
Male sex	0.90	0.84–0.96	0.002
Age, per year	1.00	0.99–1.00	0.075
Self-reported comorbidities			
Respiratory disease	1.17	1.05–1.30	0.003
Cardiac disease	1.35	1.17–1.56	< 0.001
Diabetes mellitus	1.51	1.25–1.84	< 0.001
Obesity	1.23	1.09–1.38	0.001
Other illness	1.27	1.15–1.40	< 0.001
**Country of residence**			
Peru	Reference	—	—
Chile	0.86	0.77–0.96	0.009
Paraguay	0.62	0.53–0.72	< 0.001
Mexico	0.98	0.85–1.14	0.832
Colombia	0.80	0.56–1.15	0.233
Bolivia	1.16	1.02–1.31	0.025
Panama	0.99	0.80–1.23	0.931
Ecuador	1.10	0.95–1.27	0.214
Costa Rica	0.65	0.47–0.91	0.012
El Salvador	1.16	0.99–1.36	0.075
Honduras	1.03	0.80–1.33	0.813
Guatemala	1.06	0.74–1.51	0.752

aPR: adjusted prevalence ratio; CI: confidence interval. The model was adjusted for sex, age, self-reported respiratory disease, cardiac disease, diabetes mellitus, obesity, other illness, and country of residence.

High perceived consequences of contagion was defined as a score of 9–15 on the three-item consequences domain.

Sensitivity analyses produced estimates that were materially consistent with the primary analyses. After additional adjustment for educational attainment, all individual comorbidities remained associated with a higher prevalence of high overall fatalism, with aPRs ranging from 1.17 for respiratory disease to 1.32 for diabetes mellitus (Supplementary Table S1). When the individual comorbidity indicators were replaced with a composite measure, reporting at least one comorbidity was associated with high overall fatalism (aPR 1.31, 95% CI 1.22–1.40), high concern about infection or hospitalization (aPR 1.21, 95% CI 1.14–1.29), and high perceived consequences of contagion (aPR 1.30, 95% CI 1.22–1.40; Supplementary Table S2). Excluding countries with fewer than 100 participants (*n* = 6,219) and restricting the analysis to participants reporting no previous COVID-19 diagnosis (*n* = 6,060) did not materially alter the direction or magnitude of the main associations (Supplementary Tables S3 and S4).

## Discussion

This study found that 25.4% of participants agreed or completely agreed that they could die if infected with COVID-19. Similar findings have been reported in Peru, where 26% of participants expressed the same concern [10], and in Bolivia, where 24% reported fearing death from SARS-CoV-2 infection [16]. In the United States, a study reported a positive correlation between coronavirus-related worry and associating COVID-19 with death (b = 0.43, 95% CI: 0.32–0.55, *p* < 0.001) [17]. In our study, self-reported comorbidities were consistently associated with a modestly higher prevalence of high fatalism-related outcomes, particularly for diabetes mellitus, cardiac disease, obesity, respiratory disease, and other illnesses. For example, a study in Colombia reported a 5.3% prevalence of suicide risk, which was associated with fatalistic beliefs (OR = 3.09, 95% CI: 1.53–6.27, *p* < 0.05) [11]. Taken together, these findings suggest that fatalistic perceptions during infectious disease emergencies may be relevant for mental health surveillance, although the cross-sectional design of our study precludes causal inference.

Approximately four out of ten respondents reported feeling depressed, and one out of four believed they might die if infected. These perceptions may result from pre-existing mental health conditions, anxiety, fear, and the influence of media coverage [18, 19]. To control rapidly spreading diseases with limited scientific information, it is critical to coordinate and strengthen information dissemination systems to prevent misinformation and excessive fatalism [17]. Effective interventions should include rapid diagnosis and ensure access to healthcare services for both physical and mental health needs. Integrating mental health strategies into emergency response plans can help reduce psychiatric morbidity during pandemics [8, 20].

Our findings also showed that individuals with respiratory or cardiac diseases, diabetes, obesity, and other illnesses had a higher prevalence of fatalism-related outcomes. One possible explanation is that these conditions are recognized as risk factors for COVID-19 mortality [21]. Participants with diabetes, in particular, had a higher prevalence of high concern about infection or hospitalization (aPR 1.22, 95% CI 1.03–1.46), consistent with previous research showing higher concern among diabetic individuals (54.5% vs. 36%, *p* < 0.001), which was associated with unhealthy lifestyle changes such as increased alcohol and tobacco consumption [22]. Diabetes represents a highly vulnerable population in Latin America, which may partly explain why participants with diabetes reported greater fatalistic perceptions in this study [23]. Similarly, individuals with obesity reported reduced physical activity, increased food intake, and more frequent episodes of anxiety and depression during the pandemic [24]. These results suggest that people with self-reported chronic conditions may require particular attention in risk communication and mental health responses during health emergencies.

Gender and age differences were observed, with men showing a lower prevalence of high overall fatalism and high perceived consequences of contagion. Age showed a small positive association with concern about infection or hospitalization but was not clearly associated with the other fatalism outcomes after adjustment. These results align with findings from Spain, where younger populations were most psychologically affected by the pandemic (*p* < 0.001) [25]. Nevertheless, older adults remain vulnerable to psychological consequences due to higher mortality rates [26, 27] and potential negative self-perceptions of aging, which may further impact mental health [28, 29]. Therefore, age- and sex-related findings should be interpreted in light of the young age distribution and non-probabilistic sampling of our study.

Exploratory variation was observed among the recruited country-specific subsamples. In adjusted analyses using Peru as the reference category, some country subsamples showed higher or lower prevalence estimates across the three fatalism outcomes. These patterns should not be interpreted as evidence of true differences between national populations because recruitment was non-probabilistic, country-specific sample sizes were markedly unequal, and several countries contributed fewer than 100 participants. Possible contextual explanations include variation in epidemic timing, public health responses, risk communication, health system capacity, and social conditions; however, none of these factors was measured in the present dataset. These interpretations should therefore be regarded only as hypotheses for future research rather than explanations supported by the current analysis.

Several limitations should be considered when interpreting these findings. First, the study used a non-probability online snowball sampling strategy, which limits representativeness and may have overrepresented younger, more educated, and more digitally connected individuals. Second, the distribution of participants was unequal across countries, with Peru contributing the largest proportion of the sample and some countries contributing relatively small numbers of participants. Therefore, country-specific estimates and comparisons should be interpreted as exploratory. Third, comorbidities and previous COVID-19 diagnosis were self-reported, which may have introduced misclassification. To address this concern, we performed sensitivity analyses restricted to participants reporting no previous COVID-19 diagnosis, and the main findings were materially similar. Fourth, the complete seven-item instrument was not administered, and the analysis therefore relied on a shortened study-specific measure derived from the original scale. Although the five-item overall score showed acceptable internal consistency, the domain-specific measures showed lower reliability, particularly the two-item infection/hospitalization domain. These measurement limitations reduce confidence in the secondary domain-specific estimates and may have introduced nondifferential measurement error, potentially attenuating or destabilizing associations. Accordingly, domain-specific findings should be interpreted cautiously and considered exploratory. Fifth, residual confounding remains likely because several determinants of both perceived fatalism and chronic disease status were not measured or were not available for complete adjustment. These include socioeconomic position, educational and occupational circumstances, baseline anxiety or depressive symptoms, previous mental healthcare use, intensity and framing of media exposure, local COVID-19 incidence and mortality, perceived personal risk, trust in public institutions, and geographic or financial access to healthcare. The absence of these variables may have resulted in uncontrolled or incompletely controlled confounding, and the observed associations may therefore partly reflect differences in vulnerability, information exposure, or healthcare access rather than comorbidity status alone. Finally, because of the cross-sectional design, the observed associations should not be interpreted as causal relationships.

Despite these limitations, this study has several strengths. It included a large sample of more than 6,000 participants from 12 Latin American countries during an early and highly uncertain phase of the COVID-19 pandemic. The study also evaluated fatalistic perceptions using items derived from a previously published COVID-19 fatalism scale and incorporated additional reliability estimates for the shortened version. Furthermore, the analysis used prevalence ratios rather than odds ratios, which are more interpretable for common outcomes, and included several sensitivity analyses to assess the robustness of the findings.

In this non-probabilistic sample of participants recruited in 12 Latin American countries, self-reported respiratory disease, cardiac disease, diabetes mellitus, obesity, and other illnesses were associated with a modestly higher prevalence of perceived fatalism regarding possible COVID-19 infection. The strongest associations were observed for diabetes mellitus and cardiac disease, particularly in the exploratory analysis of perceived consequences of contagion. These findings should be interpreted as exploratory and hypothesis-generating because of the cross-sectional design, self-reported exposures, potential residual confounding, and the young, Peru-predominant, non-probability sample. They should not be interpreted as nationally representative estimates or as evidence of causal relationships. Population-based and longitudinal studies are needed to clarify these associations and assess their relevance for risk communication and mental health responses during infectious disease emergencies.

## Supplementary Information

Below is the link to the electronic supplementary material.

Supplementary Material 1

## Data Availability

The datasets generated and/or analyzed during the current study are available from the corresponding author upon reasonable request, due to ethical restrictions related to participant privacy.
