# Supplementary material for: Perceived fatalism regarding possible COVID-19 infection and its association with self-reported comorbidities in 12 Latin American countries
Source: BMC Infect Dis. 2026 Jul 24;26:1852. doi: 10.1186/s12879-026-14042-7 (PMC13688888; doi:10.1186/s12879-026-14042-7)
Supplement: Supplementary file 1 [file 12879_2026_14042_MOESM1_ESM.docx]

| **Supplementary Table S1. Associations between self-reported comorbidities and fatalism-related outcomes after additional adjustment for educational attainment** | | | |
| --- | --- | --- | --- |
| Poisson regression models with log link and robust variance, n = 6,520 | | | |
|  | **aPR** | **95% CI** | **p-value** |
| **High overall fatalism** | | | |
| **Respiratory disease** | 1.17 | 1.05–1.29 | 0.004 |
| **Cardiac disease** | 1.25 | 1.09–1.44 | 0.002 |
| **Diabetes mellitus** | 1.32 | 1.08–1.60 | 0.006 |
| **Obesity** | 1.23 | 1.10–1.38 | <0.001 |
| **Other illness** | 1.26 | 1.15–1.39 | <0.001 |
| **High concern about infection or hospitalization** | | | |
| **Respiratory disease** | 1.16 | 1.06–1.28 | 0.002 |
| **Cardiac disease** | 1.12 | 0.97–1.28 | 0.113 |
| **Diabetes mellitus** | 1.22 | 1.02–1.46 | 0.029 |
| **Obesity** | 1.15 | 1.03–1.28 | 0.011 |
| **Other illness** | 1.18 | 1.08–1.29 | <0.001 |
| **High perceived consequences of contagion** | | | |
| **Respiratory disease** | 1.17 | 1.06–1.30 | 0.003 |
| **Cardiac disease** | 1.34 | 1.16–1.55 | <0.001 |
| **Diabetes mellitus** | 1.50 | 1.23–1.82 | <0.001 |
| **Obesity** | 1.23 | 1.10–1.38 | <0.001 |
| **Other illness** | 1.27 | 1.15–1.40 | <0.001 |
| aPR: adjusted prevalence ratio; CI: confidence interval. Models were adjusted for sex, age, country of residence, respiratory disease, cardiac disease, diabetes mellitus, obesity, other illness, and educational attainment. Ten participants were excluded because educational attainment was missing. Domain-specific outcomes were secondary and exploratory because of their lower reliability. | | | |

| **Supplementary Table S2. Association between reporting at least one self-reported comorbidity and fatalism-related outcomes** | | | |
| --- | --- | --- | --- |
| Poisson regression models with log link and robust variance, n = 6,530 | | | |
|  | **aPR** | **95% CI** | **p-value** |
| **High overall fatalism** | | | |
| **At least one self-reported comorbidity** | 1.31 | 1.22–1.40 | <0.001 |
| **High concern about infection or hospitalization** | | | |
| **At least one self-reported comorbidity** | 1.21 | 1.14–1.29 | <0.001 |
| **High perceived consequences of contagion** | | | |
| **At least one self-reported comorbidity** | 1.30 | 1.22–1.40 | <0.001 |
| aPR: adjusted prevalence ratio; CI: confidence interval. The exposure was a composite indicator defined as reporting at least one of the following conditions: respiratory disease, cardiac disease, diabetes mellitus, obesity, or another illness. Models were adjusted for sex, age, and country of residence. The reference category was reporting none of the assessed comorbidities. Domain-specific outcomes were secondary and exploratory because of their lower reliability. | | | |

| **Supplementary Table S3. Associations between self-reported comorbidities and fatalism-related outcomes after excluding countries with fewer than 100 participants** | | | |
| --- | --- | --- | --- |
| Poisson regression models with log link and robust variance, n = 6,219 | | | |
|  | **aPR** | **95% CI** | **p-value** |
| **High overall fatalism** | | | |
| **Respiratory disease** | 1.15 | 1.04–1.28 | 0.010 |
| **Cardiac disease** | 1.26 | 1.09–1.46 | 0.001 |
| **Diabetes mellitus** | 1.30 | 1.06–1.59 | 0.010 |
| **Obesity** | 1.26 | 1.12–1.41 | <0.001 |
| **Other illness** | 1.27 | 1.15–1.39 | <0.001 |
| **High concern about infection or hospitalization** | | | |
| **Respiratory disease** | 1.13 | 1.02–1.25 | 0.020 |
| **Cardiac disease** | 1.12 | 0.97–1.29 | 0.113 |
| **Diabetes mellitus** | 1.22 | 1.01–1.47 | 0.034 |
| **Obesity** | 1.17 | 1.04–1.30 | 0.007 |
| **Other illness** | 1.18 | 1.08–1.30 | <0.001 |
| **High perceived consequences of contagion** | | | |
| **Respiratory disease** | 1.17 | 1.05–1.30 | 0.003 |
| **Cardiac disease** | 1.35 | 1.17–1.57 | <0.001 |
| **Diabetes mellitus** | 1.51 | 1.24–1.84 | <0.001 |
| **Obesity** | 1.24 | 1.10–1.39 | <0.001 |
| **Other illness** | 1.26 | 1.14–1.39 | <0.001 |
| aPR: adjusted prevalence ratio; CI: confidence interval. Models were adjusted for sex, age, country of residence, respiratory disease, cardiac disease, diabetes mellitus, obesity, and other illness. Colombia (n = 73), Costa Rica (n = 99), Honduras (n = 95), and Guatemala (n = 44) were excluded. Domain-specific outcomes were secondary and exploratory because of their lower reliability. | | | |

| **Supplementary Table S4. Associations between self-reported comorbidities and fatalism-related outcomes among participants reporting no previous COVID-19 diagnosis** | | | |
| --- | --- | --- | --- |
| Poisson regression models with log link and robust variance, n = 6,060 | | | |
|  | **aPR** | **95% CI** | **p-value** |
| **High overall fatalism** | | | |
| **Respiratory disease** | 1.15 | 1.03–1.28 | 0.012 |
| **Cardiac disease** | 1.26 | 1.09–1.46 | 0.002 |
| **Diabetes mellitus** | 1.29 | 1.06–1.57 | 0.011 |
| **Obesity** | 1.27 | 1.13–1.42 | <0.001 |
| **Other illness** | 1.26 | 1.14–1.38 | <0.001 |
| **High concern about infection or hospitalization** | | | |
| **Respiratory disease** | 1.15 | 1.04–1.27 | 0.008 |
| **Cardiac disease** | 1.13 | 0.98–1.30 | 0.098 |
| **Diabetes mellitus** | 1.25 | 1.05–1.49 | 0.013 |
| **Obesity** | 1.18 | 1.05–1.32 | 0.004 |
| **Other illness** | 1.15 | 1.04–1.27 | 0.004 |
| **High perceived consequences of contagion** | | | |
| **Respiratory disease** | 1.14 | 1.02–1.27 | 0.021 |
| **Cardiac disease** | 1.36 | 1.18–1.58 | <0.001 |
| **Diabetes mellitus** | 1.53 | 1.26–1.85 | <0.001 |
| **Obesity** | 1.26 | 1.11–1.41 | <0.001 |
| **Other illness** | 1.26 | 1.14–1.40 | <0.001 |
| aPR: adjusted prevalence ratio; CI: confidence interval. Models were adjusted for sex, age, country of residence, respiratory disease, cardiac disease, diabetes mellitus, obesity, and other illness. The analysis was restricted to participants reporting no previous COVID-19 diagnosis. Domain-specific outcomes were secondary and exploratory because of their lower reliability. | | | |
